# Supplementary material for: Influence of parental anthropometry and gestational weight gain on intrauterine growth and neonatal outcomes: Findings from the MAI cohort study in rural India
Source: PLOS Glob Public Health. 2023 Aug 28;3(8):e0001858. doi: 10.1371/journal.pgph.0001858 (PMC10461821; doi:10.1371/journal.pgph.0001858)
Supplement: S1 File — (PDF) [file pgph.0001858.s002.pdf]

ETHICS COMMITTEE JEHANGIR CLINICAL DEVELOPMENT CENTRE PVT. LTD.
Jehangir Hospital Premises, 32 Sassoon Road, Pune - 1
Tel.:+91-20-67268821/67268804 Fax : 26059319

To,

02 March 2020

Dr. Anuradha Khadilkar

Principal Investigator

Hirabai Cowasji Jehangir Medical Research Institute

Lower Ground Floor, Block V,

Jehangir Hospital

32, Sassoon Road,

Pune- 411001.

Study title: A Study Assessing the Patterns and Determinants of Gestational Weight Gain in Pregnant Women and the Impact of Gestational Weight Gain on Maternal and Neonatal Outcomes.

Subject: Initial EC approval for the above referenced study.

Dear Dr. Anuradha Khadilkar

The Institutional Ethics Committee, Jehangir Clinical Development Center Pvt. Ltd. has reviewed and discussed your Submission letter dated 08 Feb 2020 to conduct the study entitled "A Study Assessing the Patterns and Determinants of Gestational Weight Gain in Pregnant Women and the Impact of Gestational Weight Gain on Maternal and Neonatal Outcomes." the following documents were reviewed and discussed at the Ethics Committee meeting held on 22 Feb 2020.

The below mentioned documents were approved by EC:-

- Study Thesis.
- Informed Consent Form English, Marathi and Hindi.

The below documents were notified by EC:-

- CV and MRC of Investigator
- Translation Certificates of ICFs.

Scanned with CamScanner

The meeting was conducted at JCDC Conference hall, between 01.15pm to 2:30pm. The members who attended the meeting where your proposal was discussed are as below:

LIST OF MEMBERS WHO ATTENDED THE MEETING:

MEMBER NAME	OFFICE ON EC	ORGANIZATION TITLE/HIGHEST DEGREES EARNED	SEX	PRIMARY SCIENTIFIC OR NONSCIENTIFIC SPECIALTY	AFFILIATION WITH INSTITUTION (S) (YES/NO; IF YES, WHICH ONE(S))
Dr. Ravindra Ghooi	Chairperson	Ph.D. Medicine, M.Sc (Pharmacology), B.Sc	M	Scientific	No
Mrs. Neelambari Bhosale	Member Secretory	MBA, B.Pharm	F	Scientific	Yes
Dr. Piyush Chaudhari	Clinician	MBBS, DNB	M	Scientific	Yes
Mr. Prakash Shinde	Lay Member	B.E.	M	Non-Scientific	No
Dr. Mohini Barde	Basic Medical Scientist	MD in Clinical Pharmacology & Therapeutics , MBBS	F	Scientific	No
Mrs. Esther Chopra	Social Scientist	Ph.D, M.A., B.A in Pholosophy.	F	Non-Scientific	No
Dr. Deepa Paturkar	Legal expert	Ph.D., L.L.M., L.L.B.	F	Non- Scientific	No

Scanned with CamScanner

We approve the study to be conducted in its presented form.

The Ethics Committee Biomedical and Health Research, Jehangir Clinical Development Centre Pvt. Ltd. expects to be informed about the progress of the study, any SAE occurring in the course of the study, any changes in the protocol and patient information/informed consent and asks to be provided a copy of the final report.

We hereby confirm that The Ethics Committee Biomedical and Health Research, Jehangir Clinical Development Centre Pvt. Ltd. is organized and operates as per New Drug and Clinical Trial Rules 2019 and applicable regulations.

The approval of the study is valid till 01 March 2021.

Yours Sincerely,

Signature :

Name : Dr. Ravindra Ghooi

Chairperson

Ethics Committee Jehangir Clinical Development Centre Pvt. Ltd.

EC registration No: ECR/352/Inst/MH/2013/RR-19

NABH Certification No: EC-CT-2018-0023.

Scanned with CamScanner
